# Photothermal therapeutic application of gold nanorods-porphyrin-trastuzumab complexes in HER2-positive breast cancer

**DOI:** 10.1038/srep42069

**Published:** 2017-02-03

**Authors:** Xinmei Kang, Ximing Guo, Weiwei An, Xingjian Niu, Suhan Li, Zhaoliang Liu, Yue Yang, Na Wang, Qicheng Jiang, Caichuan Yan, Hui Wang, Qingyuan Zhang

**Affiliations:** 1 Department of Medical Oncology, Cancer Hospital of Harbin Medical University, Harbin 150081, Heilongjiang, China; 2 School of Life Science of Technology, Harbin Institute of Technology, Harbin 150081, Heilongjiang, China; 3 Institute of Cancer Prevention and Treatment, Harbin Medical University, Harbin 150081, Heilongjiang, China; 4 Heilongjiang Academy of Medical Sciences, Harbin 150081, Heilongjiang, China

## Abstract

Gold nanorods are effective photothermal agents in diagnosis and treatment of cancer due to their specific near-infrared laser absorption. However, tumor photothermal therapy by nanorods alone is lack of targeting. Here, we described a novel nanocomplex made up of gold nanorods, porphyrin, and trastuzumab, called TGNs and investigated the TGN-mediated photothermal therapy as a potential alternative treatment of targeting HER2-positive breast cancers. By conjugating trastuzumab and porphyrin to the surface of gold nanorods, we have increased the targeting specificity and amplified the detecting effectiveness at the same time. TGN-mediated photothermal ablation by near-infrared laser led to a selective destruction of HER2-positive cancer cells and significantly inhibited tumor growth in mouse models bearing HER2 over-expressed breast cancer xenograft with less toxicity. Moreover, TGNs provided better therapeutic efficacy in comparison with the conventional molecule targeted therapy. Our current data suggest a highly promising future of TGNs for its therapeutic application in trastuzumab-resistant breast cancers.

At present, breast cancer is one of the most common malignant tumors in women globally^1^. Epidermal growth factor 2 receptor (Erb2 or HER2) is expressed in about 20–25% of human breast cancer, which is considered to be aggressive with poor prognosis^2^. Trastuzumab (Herceptin/Herclon) is an FDA-approved medicine which is developed as an effective therapeutic antibody against HER2-positive breast cancer^3^. Although remarkable improvement has been observed in the survival of patients in recent years, the development of drug resistance has limited its clinical application. A variety of mechanisms have been proved to contribute to the development of trastuzumab resistance, including over-expression of insulin-like growth factor 1 (IGF-1)^4^, glycoprotein MUC4^5^, Notch receptors^6^, and the activation of signaling pathways, such as PI3K/Akt/mTOR pathway^7^. Although a number of explorations have been performed and expected to find a better way to treat HER2 positive breast cancers, resistance still occurs in many cases^8^. Photothermal therapy is developing rapidly as a new and non-invasive treatment and has been widely applied in a variety of malignant solid tumors^9^,^10^,^11^. This sets the stage for using effective photothermal therapeutic strategies to treat trastuzumab-resistance breast cancers, for instance, gold nanoparticle-mediated photothermal therapy.

The design and construct of novel gold nanoparticles containing multiple functionalities make them possible to become a powerful tool in bio-imaging, cancer targeting, and cancer therapy^12^,^13^,^14^. Gold nanoparticles offer several potential advantages in targeting cancer cells for their strong absorbing and scattering properties^15^. The photothermal features of gold nanoparticles are generated from their properties of absorbing the near-infrared (NIR) light at the plasmon resonant wavelength and then transforming light energy into heat energy. This kind of heat energy can produce specific hyperthermia within the local part, ultimately leading to cell death^16^. For thermal applications, several types of gold nanoparticles have been performed to target cancer cells, such as gold nanoshells^11^,^17^,^18^, hollow gold nanospheres^19^, nanocages^20^, and nanorods^21^,^22^. In this research, we constructed a novel complex using gold nanorods (GNRs) coated with porphyrin as well as anti-HER2 antibody (trastuzumab) called GNRs-porphyrin-trastuzumab complexes (TGNs) and investigated the efficiency of this complex in targeting and photothermally ablating of HER2-positive breast cancer *in vitro* and *vivo*.

Comparing with other available gold nanoparticles, anisotropic GNRs exhibit characteristic capacities to target tumor cells specifically. The unique properties of nanorods are due to their wavelength tunability of the plasmon resonances in the red-NIR spectral region combined with the strong NIR extinction cross-sections^21^. When changing the nanorods aspect ratio, the plasmon resonance can be regulated easily, rendering imaging of multiple biomarkers at the same time^23^. Another character of GNRs is that they are easy to be modified^21^. Substances such as polymers, antibodies, small molecules, and DNA can be conjugated onto the surface of GNRs to enhance targeting specificity. Previous studies have successfully fabricated the GNRs conjugated with epidermal growth factor receptor (EGFR), which induced surface-enhanced Raman scattering on EGFR over-expressing cancer cells^24^,^25^. Here, we chose to bind trastuzumab onto the GNRs surface in order to target HER-2 over-expressing breast cancers specifically.

As the research moves along, limitations of the GNR- complexes are arisen since they generally lack imaging functions, and additional contrast agents are usually needed for further investigation. Thus, effective agents with intrinsic luminous capacities are strongly required for fluorescence imaging. Porphyrins can be used as a multimodality nanoprobe in the therapeutic application, and have been widely employed in near-infrared fluorescence imaging due to their luminous characteristics^26^. They can be easily detected in bodies, especially in tumors, for their preferential accumulation as well as amplifiable signals at tumor sites^26^. To our knowledge, this is the first time using trastuzumab binding onto the porphyrin-conjugated GNRs to figure out its efficacy and the thermal ablation in HER2-positive breast cancers. Furthermore, the study will hopefully promote current diagnostic and therapeutic strategies in management of trastuzumab-resistance breast cancer.

## Results

### Synthesis of the TGNs

**Figure 1:**
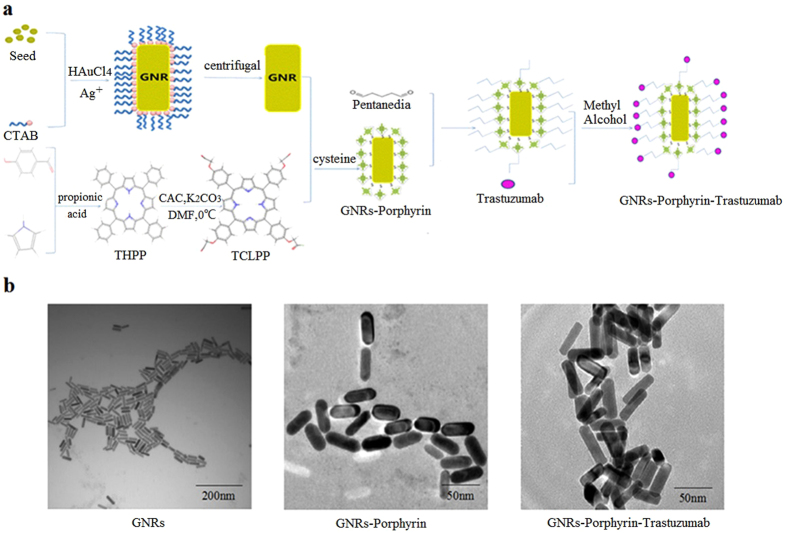
Synthesis of the TGNs. (**a**) Schematic representation of GNRs coated with porphyrin and trastuzumab. GNRs were firstly modified by porphyrin and then conjugated with trastuzumab. (**b**) TEM images of the GNRs, GNRs-porphyrin, and TGNs. TGNs show relatively irregular with wrapped layer on the surface and have a stronger intensity than GNRs and GNRs-porphyrin.

A schematic illustration of the GNRs conjugated with porphyrin and trastuzumab are depicted in Fig. 1a. GNRs were coated with porphyrin at the first step and then conjugated by trastuzumab. In order to observe the morphological characteristics of TGNs, transmission electron microscopy (TEM) was conducted to compare TGNs with GNRs and GNRs-porphyrin (Fig. 1b). The GNRs are uniform in size, with an approximately 40 nm long diameter, 10 nm short diameter, and the aspect ratios (length/width) is 4. When coated with porphyrin, no obvious change can be seen in GNRs-porphyrin compared with CTAB-coated GNRs. In comparison with GNRs, TGNs are relatively irregular, with an apparently wrapped layer on the surface. Furthermore, TGNs also show a larger intensity than GNRs-porphyrin. It could be explained by the crosslinking reaction between glutaraldehyde and the conjugates as both the aldehyde groups of glutaraldehyde have the possibility binding with GNRs-porphyrin conjugates, making the TGNs more intense as shown in the TEM images.

### Characterization of the TGNs

**Figure 2:**
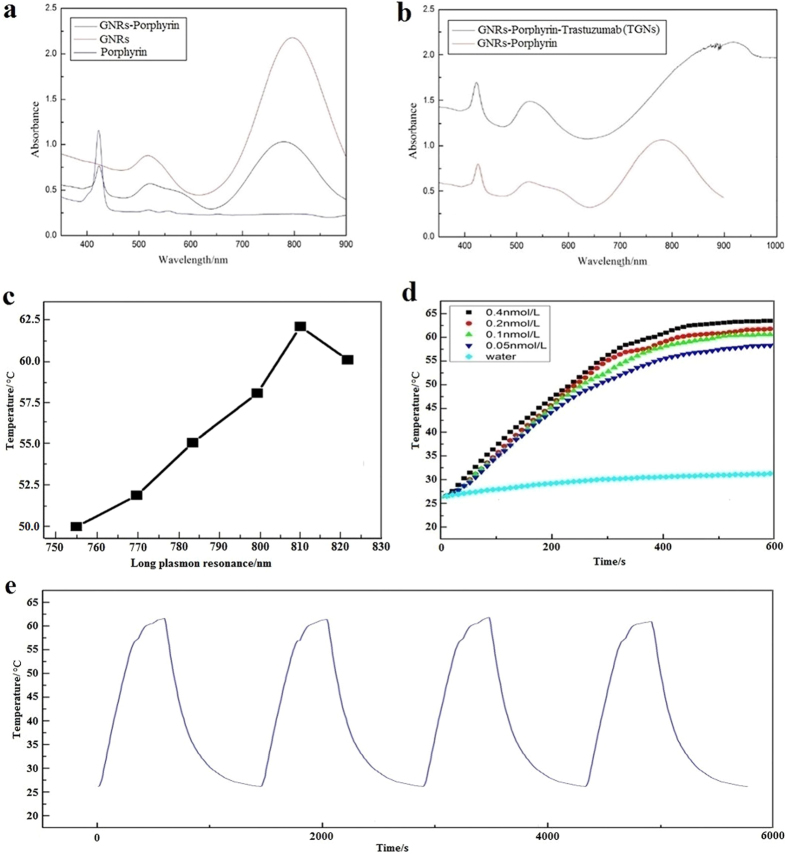
Characterization of the TGNs. (**a,b**) Absorbance spectrum of the GNRs, porphyrin, GNRs-porphyrin as well as TGNs by UV-Vis spectrophotometer. (**c,d**) The photothermal conversion of TGNs with various longitudinal absorption bands and concentrations by irradiation for 12 min with an 808 nm diode laser at a power density of 2 W/cm^2^ respectively. (**e**) Temperature difference profiles of TGNs (0.2 nmol/L) in consecutive 4 cycles of heating-cooling process.

Normalized absorption spectra of the GNRs, porphyrin, GNRs-porphyrin, as well as TGNs were obtained and shown in Fig. 2a,b. According to Fig. 2a, two absorption peaks could be seen in the GNR spectrum at 520 nm and 790 nm (red); one in the porphyrin spectrum at 423 nm (blue); as to GNRs-prophyrin conjugates, three peaks could be observed at 423 nm, 520 nm, and 800 nm from left to right (black). This data indicates that 423 nm is probably the characteristic absorbing near UV-blue region of porphyrin, while GNRs have the transverse and longitudinal peaks at 520 nm and 800 nm, respectively. After conjugation of trastuzumab, TGNs exhibit three absorption peaks at 423 nm, 520 nm and 900 nm from left to the right (black) as shown in Fig. 2b. According to the results, we believe that the 423 nm peak is the individual absorption peak of porphyrin, while the 520 nm and 900 nm peaks are transverse and longitudinal peaks of the GNRs, respectively. Comparing with GNRs-Porphyrin, the first two ultraviolet spectrum peaks of TGNs have no significant change. However, the characteristic longitudinal plasmon absorption is red shifted by over 100 nm. This red shift may be due to the crosslinking process mentioned above, in which both the aldehyde groups of glutaraldehyde can be bonded with two GNRs-Porphyrin complexes at the same time.

The photothermal conversion of TGNs with various longitudinal absorption bands was investigated by irradiation for 12 min with an 808 nm diode laser at a power density of 2 W/cm^2^ respectively, as shown in Fig. 2c. The results showed that when the absorption bands of the TGNs were between 755 nm and 810 nm, the heating rates and the constant values of temperature were increased along with the absorption peaks; while over 810 nm, the heating rates and the constant values dropped off, indicating that the longitudinal absorption bands at 810 nm of TGNs have good photothermal conversion under the present experimental conditions. Furthermore, the temperature changes in the different concentrations of TGNs at 810 nm were investigated under the irradiation by using 808 nm laser light with a power density of 2 W/cm^2^, as shown in Fig. 2d. When the highest concentration of TGNs (0.4 nmol/L) was exposed to laser for 10 min, the temperature elevation gradually reached around 63.8 °C; for the lowest detectable concentration (0.05 nmol/L), the maximum was 58 °C, also above the critical temperature to kill tumor cells. In addition, as with the increasing of the concentration, the heating rates and the constant values of temperature were also increased, far above DI water. These results consistently showed the excellent photothermal characters of the TGNs.

The stability of TGN at 810 nm was performed by the 4 cycles of temperature profiles in 0.2 nmol/L TGNs under 808 nm laser light with a power density of 2 W/cm^2^, as shown in Fig. 2e. The highest temperatures of the solution were 61.7 °C, 61.5 °C, 61.9 °C, and 61 °C in the 4 cycles respectively, and every cooling-down time was about 15 min. These results demonstrated that TGNs had very high photothermal stability, which was considered suitable for repeated experiments.

### Fluorescence microscopic images of TGN–cell binding

**Figure 3:**
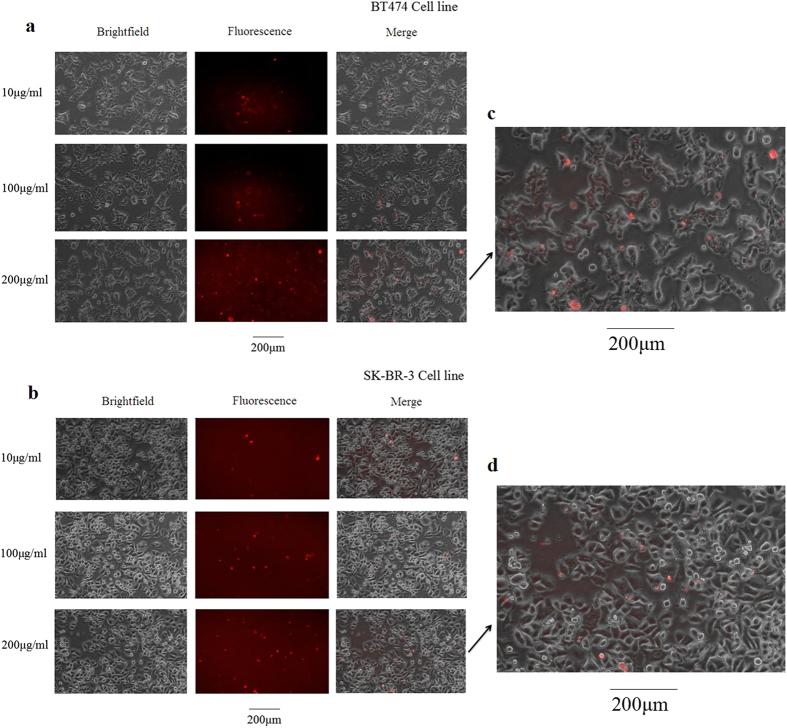
Images of TGN binding efficiency via bright and fluorescence microscopy. (**a,b**) BT474 and SK-BR-3 cell lines show great binding capacities of the conjugates, and the efficiency is positively related to the concentration of TGNs. (**c,d**) The amplified merge pictures of BT474 and SK-BR-3 cells incubated with TGNs (200 μg/ml).

The capacity of the TGNs binding onto the tumor cells was performed by fluorescence imaging. This imaging system is capable of capturing and enhancing the luminescent signature of the GNRs-porphyrin conjugates. Figure 3 represents such brightfield and fluorescent images of BT474 and SK-BR-3 cell lines incubated with TGNs. After 24 hours, both the cell lines showed significant porphyrin signals, demonstrating successful binding results. The merge images reveal the signals clearly. In order to figure out the influence of the concentration of TGNs on the binding efficiency, BT474 and SK-BR-3 cell lines were incubated with three various concentrations of TGNs. As shown in Fig. 3a,b, the higher concentration of the TGNs are used, the more conjugates can be found to bind onto the surface of either cell line. In addition, as amplified pictures shown in Fig. 3c,d, BT474 cell line showed more binding signals of TGN conjugates than SK-BR-3 cell line when incubated with 200 μg/ml of TGNs, which was consistent with the former studies that BT474 cells had more HER2-antigen binding sites than SK-BR-3 cell line^10^. Red fluorescence dots were evenly distributed on the cell surface of BT474 cell line, shown as purple-red since some of them were smaller and darker (Fig. 3c); while in SK-BR-3 cell line, fewer red dots could be seen comparing with the BT474 cell line (Fig. 3d).

### Electron microscope images in HER2-positive breast cancer cells

**Figure 4:**
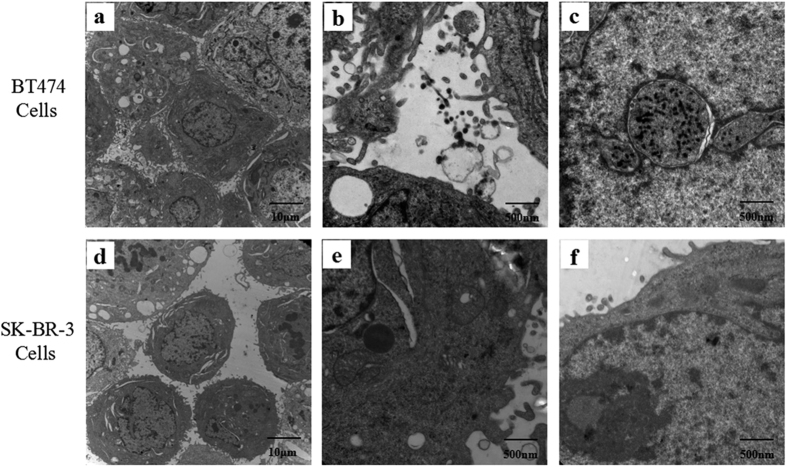
TEM images of TGNs absorption by BT474 and SK-BR-3 cells. (**a,d**) General morphology of BT474 cells and SK-BR-3 cells. (**b,c**) Images of TGNs in the intercellular space as well as the cytoplasm in BT474 cells. (**e,f**) Fewer TGNs can be seen in SK-BR-3 cells.

We also performed TEM to image TGNs in the cellular compartments and the intercellular spaces, considering the high electron density of GNRs. In electron microscopic images, GNRs in ultrathin spices appeared as little rods or cobs. Figure 4 shows the general cell morphology of BT474 and SK-BR-3 cells, the distribution of TGNs and their cellular uptake by these cells through TEM scanning. BT474 cells grow closely to each other with small intercellular space, where aggregative or dispersive TGNs can be seen in the cellular gaps as well as the cytoplasm (Fig. 4a). In higher magnification images, the location of TGNs in BT474 cells can be visualized clearly and multivesicular bodies encapsulated with nanorods can be seen in greater detail (Fig. 4b,c). TGNs are located at the intercellular spaces, especially near the cyto-membrane; some of them are engulfed by synaptic vesicles, apt to be incorporated into BT474 cells (Fig. 4b). Endocytic bodies of TGNs can be observed in the nucleus (Fig. 4c). However, the vesicles full of nanorods are invaginated from the cytoplasm. As for SK-BR-3 cells, few TGNs can be seen in any of the images (Fig. 4d–f). Comparing with the BT474 cells, SK-BR-3 cells have fewer synapses and larger intercellular spaces (Fig. 4d), only a few cobs can be observed in the synaptic vesicle or the cytoplasm (Fig. 4e,f). The results of our TEM experiments further demonstrate that BT474 cells show greater uptake activity than SK-BR-3 cells, probably because that the TGNs are mainly located at the surface of BT474 cells, which might be up taken effectively, and then carried into the cytoplasm.

### Photothermal therapeutic validity of TGNs

**Figure 5:**
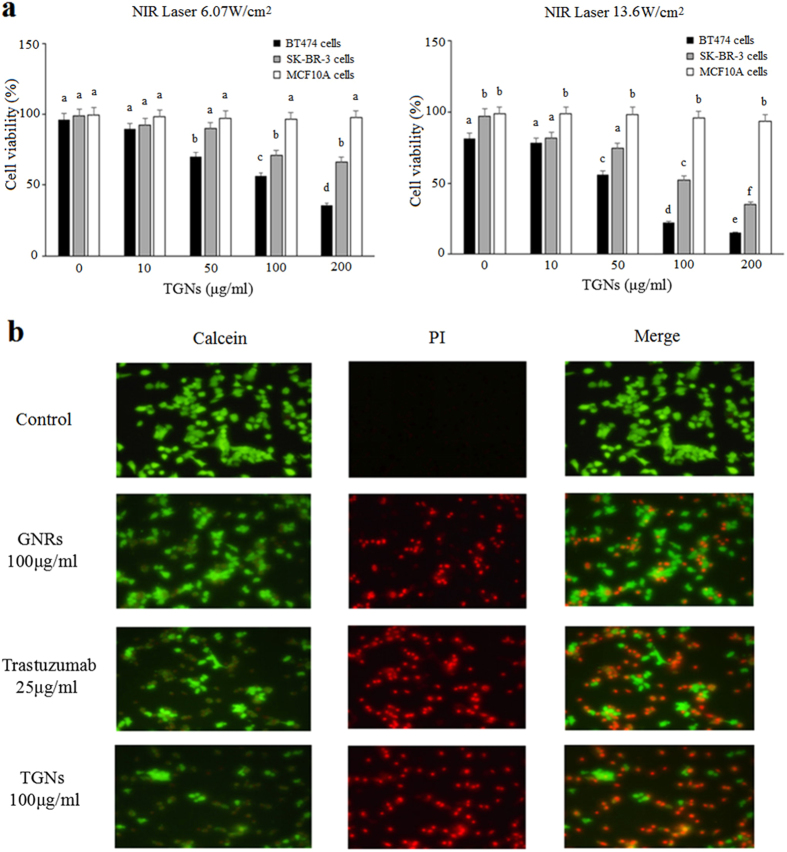
Photothermal therapeutic validity of TGNs. (**a**) CCK-8 assay of photothermal therapy of TGNs. BT474 and SK-BR-3 cells with TGNs show an incredible decrease of cell viability with the increasing concentration of the TGNs, and stronger NIR laser leads to lower viability. By comparison, no significant variation is observed in MCF10A cells. Columns and bars represent mean values ± SD. The columns labeled with no letters in common are considered significantly different (P < 0.05). (**b**) Live/dead images of BT474 cells treated with TGNs comparing with GNRs and trastuzumab. After NIR laser irradiation, TGNs is more effective than GNRs and trastuzumab, as fewer green cells and more dead cells can be observed in the last row.

To study the photothermal therapeutic effect of the TGNs on HER2-positive breast cancer and normal cells, CCK-8 assay was conducted within the BT474, SK-BR-3, and MCF10A cell lines (Fig. 5a). Each cell line was incubated with various concentrations of TGNs and then irradiated by NIR laser light with different power densities. Overall, BT474 and SK-BR-3 cell lines both showed a certain degree of decrease in cell viability after NIR laser irradiation, and 13.6 W/cm^2^ density of laser light led to collectively lower rates of cell viability than 6.07 W/cm^2^. When exposure to 6.07 W/cm^2^ irradiation, the viability rates of the BT474 cells were significantly suppressed as the dose of TGNs were increased to 50, 100, 200 μg/ml (P < 0.05), and SK-BR-3 cells were inhibited as TGNs increasing to 100 and 200 μg/ml (P < 0.05). While at 13.6 W/cm^2^ density of irradiation, BT474 cells showed dramatically lower rates in all concentrations even incubated with little TGNs (P < 0.05) and SK-BR-3 cells were suppressed with TGNs at 10, 50, 100, and 200 μg/ml (P < 0.05). Besides, the cell viability of the two cell lines at either illuminate intensity became significantly lower with the increasing of the concentrations of TGNs (P < 0.05). However, there is no obvious difference in the viability of MCF10A cells. Collectively, these results indicate that TGN-mediated photothermal therapy plays a significant role in targeting the HER2-positive breast cancer cells (particularly the BT474 cells), and probably have less toxicity to the normal cells.

To compare the photothermal therapeutic efficiency of TGNs with GNRs and the trastuzumab, live/dead imaging was applied for the treatment and control group of BT474 cell line (Fig. 5b). BT474 cell line was divided into four groups, which were incubated with culture media, GNRs, trastuzumab, and TGNs, respectively, illuminated with NIR laser light, and then stained with a Calcein/PI mixture. Live cells were stained green by calcein, and dead cells were stained red by PI. As described in Fig. 5b, the control group with culture media alone did not show any evidence of cell death, while the treatment groups with either of GNRs, trastuzumab or TGNs revealed a clearly defined area of cell death on the live/dead images. Comparing with GNRs or trastuzumab, the laser ablation of TGNs resulted in fewer green cells, leading to more dead cells which were shown in the last row. The images suggest that TGNs behave more effectively in targeting the HER2-positive cells, which makes it possible to treat trastuzumab-resistant breast cancer.

### Fluorescence imaging and photothermal therapeutic efficacy of TGNs in mouse models

**Figure 6:**
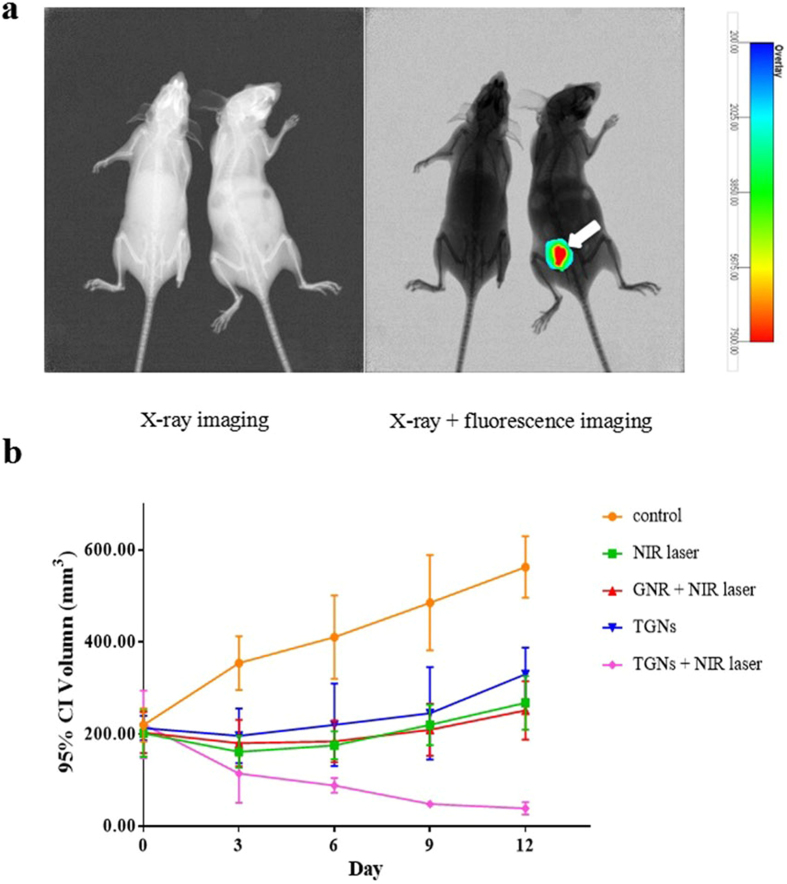
Photothermal therapeutic efficacy of TGNs in mouse models. (**a**) Representative *in vivo* X-ray and X-ray/fluorescence images of nude mice after implantation of BT474 cells (the right mouse) and MDA-MD-231 cells (the left mouse). The fluorescence signals came from the porphyrin of TGN-complexes. The white arrow indicates the tumor site. (**b**) Photothermal therapeutic effect of TGNs combined with photothermal therapy. Tumor volume of nude mice bearing BT474 breast cancer xenografts treated with TGNs once per week followed by NIR irradiation decreased significantly in comparison with the TGN only group, NIR irradiation only group, GNRs plus NIR laser irradiation group and control group.

In order to investigate the accumulation of TGNs in mice bearing with HER2-positive breast cancers, we established mouse models implanted BT474 cells (Fig. 6a, the right mouse) and MDA-MB-231 cells (Fig. 6a, the left mouse) respectively. X-ray and X-ray/fluorescence images (Fig. 6a) have shown that TGNs displayed significantly higher tumor accumulation in the BT474 breast cancer xenograft mouse, while no fluorescence signal could be observed in MDA-MB-231 xenograft mice, demonstrating that TGNs would specifically locate and target the HER2-positive breast cancer *in vivo*.

To further evaluate the effectiveness of TGNs combined with photothermal therapy in mice bearing BT474 breast cancer xenografts, we compared the therapeutic efficacy of TGNs followed by NIR laser light with TGNs only group, NIR laser irradiation only group, as well as the group of injecting GNRs followed by NIR laser irradiation, as shown in Fig. 6b. TGN-mediated photothermal therapy could significantly inhibit tumor growth in comparison with other groups. When the xenograft mice were left untreated, the tumor volumes continued to increase over time. Using TGNs without NIR laser light inhibited tumor growth initially, however, the volumes of tumor increased rapidly at extended time points. The results of using GNRs plus NIR laser group were similar to the NIR laser group, that the tumor volumes were decreased at the beginning, while presented a gradual increase followed by the days. This was probably because that the GNRs alone could not accumulate in the tumor sites without trastuzumab.

### Bio-distribution and toxicity of TGNs in xenograft mouse models

**Figure 7:**
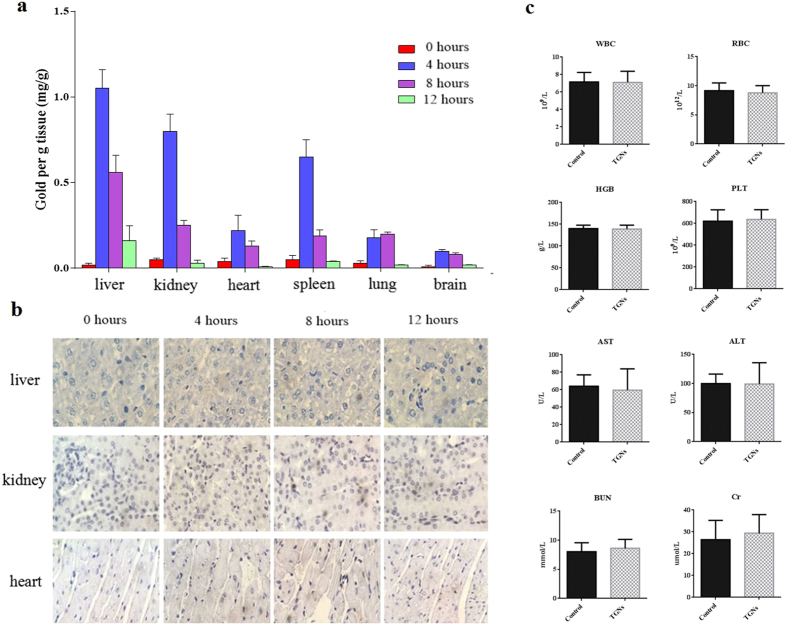
Distribution and toxicity assessment of TGNs in xenograft mouse models. (**a**) Bio-distribution of TGNs at 0, 4, 8, and 12 hours in liver, kidney, heart, spleen, lung and brain in mice calculated as mg Gold per gram tissue. (**b**) Silver staining of tissue specimens in liver, kidney, and heart. (**c**) Representative parameters of hematology and biochemistry in the mice treated with TGNs and the control group mice. Columns and bars were expressed as the mean ± SD.

We performed a comparison of the tissue distribution at 0, 4, 8, and 12 hours after TGNs injection in tumor-bearing mice. As shown in Fig. 7a, significant accumulations of gold uptake by liver, kidney, and spleen were noted in comparison with other tissues (P < 0.05), suggesting that metabolic TGNs were more likely excreted via hepatobiliary and urinary systems. In addition, 4 hours after TGNs administration resulted in significant accumulation of gold in all tissues than 0, 8, and 12 hours after TGNs injection (P < 0.05), suggesting little accumulation of TGNs in the organs of mouse models.

Silver staining of tissue specimens in liver, kidney, and heart was further conducted to visually analyze the distribution of TGNs in mouse models (Fig. 7b). After silver enhancement, the TGNs were depicted as little black dot. As shown in Fig. 7b, more TGNs could be seen in the tissue biopsies of liver and kidney, while fewer in the heart. Consistent with the quantitative results, at 4 hours after TGNs injection, more TGNs could be observed in the organs of mice, however, the expression of TGNs decreased significantly after 8 and 12 hours, further demonstrating the commendable usability of TGNs *in vivo*.

Hematological and biochemical assessments of the xenograft mice were conducted to evaluate the toxic effects of TGNs *in vivo*. Several representative parameters of hematology and clinical chemistry were shown in Fig. 7c. Serum ALT and AST levels were used as liver function biochemical indicators, and BUN, Cr were used as the renal function indicators. In the range of effective doses of TGNs, no significant differenceof all the above-mentioned parameters was found between the TGNs-treated mice and control mice (P > 0.05), which suggests that the treatment of TGNs showed less toxicity to blood picture, liver function, and renal function in xenograft mouse models.

## Discussion

As is known to all, GNRs have been proven to be promising tools in their application of diagnostic imaging, biosensing, drug delivery, and treatment^12^. GNR-mediated photothermal therapy is a novel non-invasive experimental approach and is recognized as an important therapeutic strategy for specifically targeting tumor cells. The objective of this study was to fabricate a novel trastuzumab-conjugated GNRs coated with porphyrin and determine whether it could selectively and effectively target as well as destroy HER2-positive breast cancers *in vitro* and *vivo*, in order to explore new strategies of treating trastuzumab-resistant breast cancers.

In the past decade, plenty of studies have investigated gold nanoparticles in various fields due to their biocompatibility and photo-optical distinctiveness^12^. Recent advances in the therapeutic targeting use of gold nanoparticles mostly focus on their application as protein and peptide platforms aiming at delivering medicine to tumor sites or lesion areas, and several platforms have demonstrated a great promise^27^,^28^,^29^. A number of studies have shown that antibodies^29^ and viral vectors^30^ conjugated to gold nanoparticles could successfully be applied in specific photothermal therapy. Besides, HER2-targeted gold nanoshells^10^,^31^,^32^ and nanocages^33^ could be effectively bound onto HER2 over-expressed tumor cells. When the cells were irradiated by NIR laser light, cell death could be observed. Moreover, Carpin and colleagues^10^ have investigated the impact of anti-HER2-conjugated silica-gold nanoshell on trastuzumab-resistant breast cancer cell lines, and they reported that these nanoshell complexes could effectively target trastuzumab-resistant cells and induce laser ablation, thus providing bright hope of using nanomaterials treating drug-resistance cancer patients.

In this study, we developed a novel nanocomplex consisting of GNRs, porphyrin, and trastuzumab. GNRs have been drawn great attention in recent years, which arise from their distinctive properties such as adjustable aspect ratio, high synthetic yields, size monodispersity, and easy surface modification^21^. Researchers are able to design the size and shape of the nanorods in accordance with the need of the near-infrared wavelength. It becomes feasible to construct GNRs more precisely so that optical transmission is optimal for imaging. Huang *et al*.^34^ have shown that GNRs are effective as photothermal agents due to their specific NIR absorption band in the longitude. However, tumor photothermal therapy by GNRs alone is lack of targets. In our research, we designed anti-HER2 antibody (trastuzumab) conjugated to GNRs in order to improve the targeting specificity. In addition, this is the first time using porphyrin conjugating onto GNRs. Porphyrin is an excellent tumor therapeutic photosensitizer and contrast agents in medical imaging and has been widely applied in diagnosis and treatment of cancer. Based on its good fluorescence effect and preferential accumulation in tumor, the TGNs can be detected easily at cancerous sites without the need of other contrast agents. It’s very beneficial to investigate the circumstances of targeting and detection effectiveness of TGNs by observing the fluorescence density of porphyrins. Besides, it is possible to detect the accurate location of the tumors even if the sites are too tiny to be visible with naked eyes. Thus, by using TGNs, the targeting efficacy might be enhanced, and the detection might be amplified at the same time. In our research, we designed GNRs of 10 × 40 nm that preferentially absorb NIR light at around 810 nm region of the spectrum. Furthermore, we successfully conjugated porphyrin and trastuzumab onto the GNRs. In order to determine the practical application of the TGNs, we evaluated their binding efficiency and photothermal destruction *in vitro* and *vivo*.

According to our results, TGNs has shown great binding capacities with HER2-positive breast cancers, and TGN-mediated photothermal therapy has led to a selective destruction of HER2 positive breast cancer cells, which is similar to the results of former studies^10^,^35^. Although it seems like gold nanoparticle-mediated photothermal therapy was not available to inhibit tumor growth in some previous studies^36^,^37^, in our study, tumor volumes of mouse models were decreased dramatically treated with TGNs plus laser illumination. This probably contributed to the distinctive characteristics of the GNRs conjugated with trastuzumab, which not only enhanced TGNs accumulation in tumor sites but combined photothermal therapy with targeting chemotherapy^36^.

Since GNRs provide the capability of absorbing the laser energy and transforming it to heat, the requirements of the irradiating energy can be lower in comparison with the conventional hyperthermia treatments. However, previously published literature has demonstrated several shortcomings of GNRs bioconjugates, among which toxicity is still a significant issue^38^. Nanorods themselves also accumulate in normal tissues, which may lead to a certain extent of normal cell death by photothermal therapy. For this reason, we conducted toxicity assays of TGNs *in vitro* and *vivo* and the results indicated that TGN-mediated cell destruction by laser irradiation had relatively less toxicity to the normal cells and did not accumulate in the bodies or affect the biological activities of mouse models in the range of effective doses. More importantly, TGNs provide better therapeutic efficacy comparing with trastuzumab, probably due to their combining characteristics of specific targeting and photothermal therapeutic effects. Hence, it’s possible to use this anti-HER2-nanorod conjugates to treat drug-resistant breast cancers.

For both BT474 and SK-BR-3 cell lines, the capacity of TGNs binding seems to correlate with TGN concentrations. However, SK-BR-3 cell line shows lower cellular uptake and less binding efficacy of TGNs than the BT474 cell line, and the data from CCK-8 assay demonstrates that the cell inhibition effect of thermal therapy on SK-BR-3 cell line is weaker than BT474 cells. This might be due to the differences in biological characteristic and the thermal sensitivity to NIR laser between the two cell lines^10^. As shown in the images of the cellular morphology through TEM, BT474 cells contain plenty of synapses, which make the cells absorb little substances easily. Moreover, they tend to grow in conglomerates, while the SK-BR-3 cells grow in segregation, leading to different heating profiles between the two cell lines. Another possible contributing factor is that BT474 cells are more sensitive to the photothermal therapy, since they are able to bind with more TGNs than SK-BR-3 cells. As the photothermal effects of the TGNs were positively related to their concentrations, it’ not difficult to explain the better photothermal therapeutic outcomes of TGNs in BT474 cell line in comparison with SK-BR-3 cells. Generally speaking, all the factors, such as the concentration of nanoparticles, the nanorods binding efficacy, the laser intensity profile, the characteristic or thermal sensitivity of tumor cells should be considered in the TGN-mediated treatment of trastuzumab-resistant breast cancers.

In conclusion, our current data underlines several advantages of TGNs in targeting HER2-positive breast cancers and exhibits promising anti-tumor activity *in vitro* and *vivo*. Porphyrin and trastuzumab conjugating allows easy nanocomplex detection and specific binding onto HER2 over-expressed cell surface receptors. NIR laser irradiation might lead to photothermal therapeutic results without damage to the surrounding tissue and the biological activities of mouse models. More importantly, TGNs target HER2-positive cells more specifically than the conventional molecule targeted therapy, making them possible to treat drug-resistant breast cancer. This is the first demonstration of a successful combination of GNRs, porphyrin and trastuzumab, which provide a possibility for the treatment of trastuzumab-resistant breast cancers as well as other HER2-positive tumor types in clinics.

## Matetrials and Methods

### Breast cancer cell lines

Two breast cancer cell lines over-expressing different levels of HER2 receptors were chosen for this study: BT474 and SK-BR-3. We used MDA-MB-231 breast cancer cell line as the HER2-negative control group. The immortalized mammary epithelial cell line (MCF10A), which is often regarded as a relative normal mammary epithelial cell line, was also used as a control. BT474, SK-BR-3, and MDA-MB-231 cell lines were obtained from Cell Bank, Chinese Academy of Sciences, Beijing, China. MCF10A cell line was obtained from Cell Culture Center, Institute of Basic Medical Sciences Chinese Academy of Medical Sciences, Beijing, China. BT474 cells were cultured in RPMI1640 medium supplemented with 10% fetal bovine serum (FBS), 1% penicillin-streptomycin, 10 μg/ml insulin, and 0.11 g/L Sodium Pyruvate. SK-BR-3 cells were cultivated in DMEM medium supplemented with 10% FBS, 1% penicillin-streptomycin, and 2 mM L-glutamine. MDA-MB-231 cells were cultured in L15 medium containing 10% FBS and 1% penicillin-streptomycin. MCF10A cells were grown in DMEM/F12 (1:1) supplemented with 5% horse serum, 10 μg/ml insulin, 20 ng/ml EGF, 100 ng/ml cholera toxin and 0.5 μg/ml hydrocortisone. All these cell lines were incubated at 37 °C in a humidified chamber with 5% CO_2_ and cultured in exponential growth phase by subcultivation.

### GNRs fabrication

GNRs were fabricated by improved seed-mediated growth methods as previously reported^39^,^40^. Briefly, 250 μL of 0.5 mM HAuCl_4_ was added to 7.5 ml of 0.1 M cetyltrime thylammonium bromide (CTAB) water solution. Following this, 60 μL of 10 mM ice-cold NaBH_4_ was added to the reaction mixture with gentle shaking for 2 min at 25 °C to make seed solution. In parallel, GNR growth solution was established as follows. 100 μL of 4 mM AgNO_3_ was added to the mixture of HAuCl_4_ (425 μL) and CTAB (10 mL) water solution. Then, 67 μL of 78.8 mM ascorbic acid was added to the solution as a moderate reducing agent. Afterward, the seed solution was mixed with the GNR growth solution which was stirred gently until it was clear. Then the solution was kept in 27–28 °C water overnight. After the GNRs had been completely synthesized, they were removed by centrifugation at 10000 r/min for 20 min. Finally, the GNRs was dispersed in deionized water and stored at 4 °C.

### Synthesis of TGNs by conjugating porphyrin and trastuzumab onto GNRs

Porphyrin prepared for conjugation was fabricated as follows. Firstly, 5,10,15,20-tetrakis(4-hydroxyphenyl)porphyrin (THPP) was synthesized by the method of Alder-Longo^41^, and then 70 mg of THPP was added to the mixture composed by 5 g of K_2_CO_3_ and 3 ml of chloroacetyl chloride, which was dissolved in N,N-Dimethylformamide (DMF). After separation, desiccation, and chromatography, 5,10,15,20-tetra-kis-(4-chloro-tetra-phen-yl)porphyrin (TCLPP) was synthesized, and 50 mg of it was mixed with 3 g of K_2_CO_3_, 121 mg of cysteine, and 50 ml of methanol solution. At 65 °C, the mixture was kept for 12 hours to react completely. After removing the K_2_CO_3_, thiolated-porphyrin were fabricated through Au-S bond.

GNRs-porphyrin was generated using above products. Briefly, 10 ml of GNRs water solution and 5 mg of thiolated-porphyrin were dissolved in the 20 ml of methanol solution. After 12–14 hours, the reaction mixture was centrifugated at 8000 rpm for 20 min. Then, the GNRs-porphyrin conjugate was dispersed in 20 ml of methanol solution. TGNs were fabricated by the method of glutaraldehyde crosslinking^42^. At first, 20 ml methanol solution full of GNRs-porphyrin and 2–3 ml glutaraldehyde were mixed and stirred for 5 min. Then the solution was also centrifugated at 8000 rpm for 20 min. After the deposit was dispersed into methanol solution, 0.5 ml of trastuzumab (Roche Pharma(schweiz)Ltd) was added and incubated at 4 °C for 12 hours. After 3 times of centrifugation and dispersion, TGNs were finally fabricated. The dimension of the conjugates was measured by transmission electron microscopy (TEM) (Hitachi H-7650, Japan).

### Characterization of TGNs

We used UV-Vis spectrophotometer (Cary4000, Rio-Rad) to evaluate the TGNs characteristics of absorption, extinction, and scattering coefficients. To further study the TGNs properties of photothermal conversion, DI water (1 ml), and TGNs with various longitudinal absorption bands and concentrations were suspended in the cuvettes and irradiated with an 808 nm diode laser (Hi-Tech Optoelectronic Co., Ltd., Beijing, China) at a power density of 2 W/cm^2^ for 12 min, respectively. When the solution reached the maximum temperature, the diode laser was off for 15 min, allowed the solution cool to ambient temperature. The heating-cooling cycle was repeated three times. The temperature values were recorded every 10 sec and the curves were plotted.

### Fluorescence imaging of TGNs binding efficiency

BT474, SK-BR-3, and MCF10A cell lines were evaluated for surface labeling of HER2-targeted nanorods by employing fluorescence microscopy. All the cells were isolated respectively, washed with PBS, and seeded in 96-well plates (10^4^ cells/well) for further investigation. Next, these cells were incubated with three various concentrations of TGNs (10 μg/ml, 100 μg/ml, 200 μg/ml) for 24 hours. After incubation, the cell lines were centrifuged and washed with PBS three times in order to remove unbound TGNs and then resuspended in 0.5 ml (60 μl) appropriate media. We used the Olympus IX71 Microscope (Sinokey, Beijing, China) for imaging.

### TEM imaging of cellular uptake of TGNs by HER2-positive breast cancer cells

To observe the cellular uptake, 100 g/ml TGNs were added to the medium of BT474 and SK-BR-3 cell lines respectively and incubated at 37 °C in a 95% air and 5% CO_2_ atmosphere for 48 hours. After that, the cells were isolated, washed with phosphate buffer solution (PBS), and then fixed in 0.1 M cacodylate buffer (pH 7.2) with 2.5% glutaraldehyde, and embedded in epoxy resin. The resin block covered by the monolayer cells were cut into 70 nm ultrathin spices for TEM scanning without contrasting stain.

### CCK-8 (cell-counting kit-8) assessment of photothermal therapeutic validity by TGNs

Cells from each BT474, SK-BR-3, and MCF10A cell line were seeded in 96-well plates as describe above, and then incubated with five various concentrations of TGNs (0, 10 μg/ml, 50 μg/ml, 100 μg/ml, 200 μg/ml) for 24 hours. Next, all the cells were washed with PBS for three times. Then each cell line was divided into two groups: one group was irradiated by NIR laser at 6.07 W/cm^2^ with a 3 mm spot size for 5 min, the other was irradiated by NIR laser at 13.6 W/cm^2^ with a 2 mm spot size for 5 min. Then these cells were collected, and the viability was assessed with the CCK-8 (Dojindo, Japan). According to the protocol, 10 μL CCK-8 reagent was added to each well, and all the cells were incubated at 37 °C for 1–2 hours. The cell viability was measured. Every experiment was conducted in six parallel wells and repeated three times.

### Live/dead analysis of cell viability

To investigate the photothermal therapeutic effect of TGNs, GNRs, and trastuzumab, BT474 cells were grouped and incubated with GNRs (100 μg/ml), trastuzumab (25 μg/ml), and TGNs (100 μg/ml), respectively for 24 hours and treated as described above. Media alone was performed as a negative control. And then the cells were illuminated by NIR laser light at 13.6 W/cm^2^ with a 2 mm spot size for 3 min. Finally, the cells were stained using an Invitrogen Live/Dead viability/cytotoxicity kit (Carlsbad, CA, USA), and imaged using Olympus IX71 Microscope. Cell viability was evaluated using Calcein to stain live cells and propidium iodide (PI) to stain dead cells. A dye solution mixture containing 2 μM Calcein and 3 μM PI was prepared, and 150 μL was added to each well which was photothermally irradiated.

### Tumor imaging and photothermal therapy in xenograft mouse models

Female athymic nude mice (6 weeks of age) obtained from Shanghai Laboratory Animal Center was used for *in vivo* therapeutic studies. All animal experiments were conducted in compliance with the Guide for the Care and Use of Laboratory Animals published by the China National Institutes of Health and approved by the Animal Care Committees of Harbin Medical University, China. Nude mice were implanted subcutaneously with 1 mL of 1 × 10^7^/mLMDA-MB-231 or BT474 cells in the left mammary fat pad, respectively. Once the tumors reached 150 mm^3^ in volume, the mice were injected 10 mg/ml of TGNs (0.2 ml) intravenously through the tail vein. 6–8 hours later, mice were imaged using multi-spectral imaging systems (Carestream FX Pro, Beijing, China).

Nude mice bearing BT474 breast cancer xenografts were divided into five groups. Mice in the first group were injected with 0.2 ml of TGNs (7.5 mg/ml) via tail vein once per week and irradiated by laser light at 6.07 W/cm^2^ with a 5–10 mm spot size for 5 min; in the second group mice were treated with 0.2 ml of TGNs without NIR laser irradiation; mice in the third group were each injected with 0.2 ml of GNRs (7.5 mg/ml) and illuminated by laser light at the same density for 5 min; in the fourth group, mice were only treated with laser light; the last group of mice were untreated. Tumor size was measured every 3 days by a digital slide caliper, and the volume was calculated according to the formula: volume = 0.5 × length × width^2^.

### Bio-distribution and toxicity assessment of TGNs in xenograft mouse models

Nude mice bearing BT474 breast cancer xenografts were injected with 0.2 ml of TGNs (7.5 mg/ml) via tail vein. At 0 hours, 4 hours, 8 hours, 12 hours after injection of TGNs, we collected liver, kidney, heart, spleen, lung, and brain after the mice were sacrificed, respectively. The organs were dissected and weighed for further analysis. All the tissues (n = 3 for each group) were digested with 200 μl of nitric acid and incubated at 60 °C for 2 hours. Then the tissues were treated with 200 μl of 50% H_2_O_2_ for 2–4 hours and adjusted to a total volume of 1 mL. Gold concentration was detected using inductively coupled plasma mass spectrometry (ICP-MS, Agilent Techologies, 7500ce), and the results were presented as mg Gold per gram tissue.

Tissues from liver, kidney and heart were fixed in 10% buffered formalin over night. Samples were dehydrated, embedded and sectioned to 4 um-thick slices. The tissue sections were de-waxed with xylene and washed consecutively with 100%, 90%, 70% and 30% ethanol, and were immersed in a silver enhancement solution which is composed of equal amount of solution A and B from the Silver Enhancer Kit (Sigma-Aldrich). After rinsing, the tissue sections were fixed with 2.5% sodium thiosulfate for 3 min, immersed in Haematoxylin for 40 sec, washed with DI water, and were then mounted for bright field light imaging.

Peripheral blood samples for hematological and biochemical analysis were collected from the orbital sinus of mice (n = 10) 72 hours after injection of TGNs (7.5 mg/ml). Mice in control group (n = 10) were injected with the same volume of saline. The following parameters were measured with a Bayer^®^ Advia 120 hematology analyzer: white blood cell count (WBC), red blood cell count (RBC), hemoglobin (HGB), and platelets (PLT). The following serum parameters were measured with an Olympus^®^ AU640 clinical chemistry analyzer: aspartate aminotransferase activity (AST), alanine aminotransferase activity (ALT), blood urea nitrogen (BUN), and creatinine (Cr).

### Statistical analysis

Data from *in vitro* and *vivo* studies was evaluated by analysis of variance (ANOVA) and Student-Newman-Keuls test. All the values were expressed as the mean ± SD and P < 0.05 was considered statistically significant. The statistical analysis software performed was SPSS Version 22.0.

## Additional Information

**How to cite this article:** Kang, X. *et al*. Photothermal therapeutic application of gold nanorods-porphyrin-trastuzumab complexes in HER2-positive breast cancer. *Sci. Rep.* **7**, 42069; doi: 10.1038/srep42069 (2017).

**Publisher's note:** Springer Nature remains neutral with regard to jurisdictional claims in published maps and institutional affiliations.
