# Corrigendum: Photothermal therapeutic application of gold nanorods-porphyrin-trastuzumab complexes in HER2-positive breast cancer

**DOI:** 10.1038/srep44519

**Published:** 2017-04-11

**Authors:** Xinmei Kang, Ximing Guo, Xingjian Niu, Weiwei An, Suhan Li, Zhaoliang Liu, Yue Yang, Na Wang, Qicheng Jiang, Caichuan Yan, Hui Wang, Qingyuan Zhang

Scientific Reports 7: Article number: 4206910.1038/srep42069; published online: 02 03 2017; updated: 04 11 2017

The original version of this Article contained an error in the order of the authors, where “Xinmei Kang, Ximing Guo, Xingjian Niu, Weiwei An, Suhan Li, Zhaoliang Liu, Yue Yang, Na Wang, Qicheng Jiang, Caichuan Yan, Hui Wang and Qingyuan Zhang” was incorrectly given as “Xinmei Kang, Ximing Guo, Weiwei An, Xingjian Niu, Suhan Li, Zhaoliang Liu, Yue Yang, Na Wang, Qicheng Jiang, Caichuan Yan, Hui Wang and Qingyuan Zhang”.

In addition, the Acknowledgments section in this Article was incomplete:

“This study was supported by International Science & Technology Cooperation Program of China (No. 2012DFR30220)”.

Now reads:

“This study was supported by International Science & Technology Cooperation Program of China (No. 2012DFR30220) and the Natural Science Foundation of China (No. 81270034)”.

These errors have been corrected in the HTML and PDF versions of this Article.

